# Cryo-EM and the elucidation of new macromolecular structures: Random Conical Tilt revisited

**DOI:** 10.1038/srep14290

**Published:** 2015-09-22

**Authors:** C. O. S. Sorzano, M. Alcorlo, J. M. de la Rosa-Trevín, R. Melero, I. Foche, A. Zaldívar-Peraza, L. del Cano, J. Vargas, V. Abrishami, J. Otón, R. Marabini, J. M. Carazo

**Affiliations:** 1 National Center for Biotechnology (CSIC), c/Darwin, 3, Campus Universidad Autónoma, 28049 Cantoblanco, Madrid, Spain; 2 Universidad CEU San Pablo, Campus Urb. Montepríncipe s/n, 28668 Boadilla del Monte, Madrid, Spain; 3 Center for Biological Research (CSIC), c/Ramiro de Maeztu, 9, 28040, Madrid, Spain; 4 Escuela Politécnica Superior, Universidad Autónoma de Madrid, Campus Universidad Autónoma, 28049 Cantoblanco, Madrid, Spain

## Abstract

Cryo-Electron Microscopy (cryo-EM) of macromolecular complexes is a fundamental structural biology technique which is expanding at a very fast pace. Key to its success in elucidating the three-dimensional structure of a macromolecular complex, especially of small and non-symmetric ones, is the ability to start from a low resolution map, which is subsequently refined with the actual images collected at the microscope. There are several methods to produce this first structure. Among them, Random Conical Tilt (RCT) plays a prominent role due to its unbiased nature (it can create an initial model based on experimental measurements). In this article, we revise the fundamental mathematical expressions supporting RCT, providing new expressions handling all key geometrical parameters without the need of intermediate operations, leading to improved automation and overall reliability, essential for the success of cryo-EM when analyzing new complexes. We show that the here proposed RCT workflow based on the new formulation performs very well in practical cases, requiring very few image pairs (as low as 13 image pairs in one of our examples) to obtain relevant 3D maps.

The use of Random Conical Tilt (RCT)^1^,^2^,^3^,^4^,^5^ as a way to produce the initial volume for a subsequent projection matching refinement has been in place for over 30 years. The technique has revealed as a very useful approach, especially when little *a priori* structural information is available. A proof of this statement is the more than 1,100 citations of the 4 references above. Normally this technique is applied to stained samples, since its application in cryo-EM setups is cumbersome. As it is currently applied, the projection images in untilted micrographs are subjected to a 2D classification. Those classes with a high internal homogeneity are selected. Finally, RCT is applied to each class individually obtaining, in this way, a set of RCT models (one model per 2D class of untilted images). These models can then be combined, at least in some cases, in order to reduce the effect of the missing cone in Fourier space, which, otherwise, results in an elongation of the resolved structure along the missing direction^6^,^7^.

Mathematical developments related to RCT have concentrated on analysing the geometrical relationships between the Euler angles of the untilted and tilted micrographs^5^. However, a number of simplifications have traditionally been made, such as the omission of the explicit consideration of the shifts among projections introduced by inaccurate particle selection (the user cannot pick exactly at the particle origin in all projections) and the possible misalignment introduced by micrograph acquisition. Additionally, some particles may have been flipped within the holder and, in the current formulation, they have to be excluded from the 3D reconstruction because the current RCT formulation cannot manage them. However, we show that with the proper geometrical treatment, they can also be included in the reconstruction process and indeed provide useful structural information (note that the inclusion of mirrored images must be subjected to the consideration that partial staining may invalidate the assumption that mirrored and unmirrored images contain the same structural information, consequently, whether it is more beneficial to include them or not depend on each specific case).

In this paper we address the problems mentioned above. In this way, we propose a geometrical framework that explicitly accounts for possible shifts introduced during the picking process, angular misalignment during the micrograph scanning, as well as it allows the inclusion within the same 2D class of projections that are related by a mirror operation. Additionally, we propose to align tilted experimental projections to the class average of the corresponding untilted projections, again without intermediate steps. In this way, the 2D alignment of the tilted images becomes much more robust since the reference image to which the tilted images are aligned to has higher signal-to-noise ratio (SNR). All these changes together result in a very robust RCT algorithm capable of producing a 3D reconstruction of sufficient quality with very few image pairs (as few as 13 image pairs in one of our examples, as shown in the Results section).

## Method

All mathematical details of the geometrical relationships between the untilted and tilted projections are described in Supplementary Material. Let us, at this point, summarize their main steps, going from the more general to the more particular aspects:

•   Determination of the tilt axis: RCT requires the acquisition of two electron micrographs of the same field of view: one tilted and another one untilted. The location of the tilt axis needs to be determined in each of the two micrographs, and this is normally done by manual identification of a set of corresponding coordinate pairs in both micrographs. In Supplementary Material we provide a rigorous justification of the equation normally used in the field for this task (Eq. 37 of^5^), identifying its key assumptions. Namely: 1) that the location of the tilt axis relies heavily on the correctness of the hypothesis that the grid being visualized is flat; 2) that the untilted micrograph is perpendicular to the electron beam; and 3) that the centers of all selected particles are in the *z* = 0 plane of the grid. Note that this latter assumption could be avoided by an explicit refinement of the *z* location of each particle within the grid, which has never been attempted, probably because of the use of RCT for low resolution models only.
•   Alignment between the untilted and tilted images: RCT requires the alignment between the tilted projection of a particle and its untilted projection (Eq. (12) in the Supplementary Material). Note that the parameters describing this alignment are related to the location of the tilt axis in both micrographs (tilted and untilted) as well as a shift between the two projections. The location of the tilt axis was determined in the previous step and, to the best of our knowledge, it is not the main source of errors in RCT reconstructions. Therefore, the shift between the tilted and untilted projections is the only new parameter that needs to be estimated at this step. This shift should be zero if, for each projection pair, the user (or a program) were able to pick the tilt and untilted projections so that they were mutually centered, indeed a rather difficult task considering the low SNR and the need for comparing two images that may look quite different (micrographs have been tilted by a substantial amount). The way commonly used in the field to handle this issue is by performing an intermediate step of aligning the untilted image to a centered reference. Of course, it is essential to relate the untilted to the tilted images, and this is normally done through a stretching operation, which currently requires the additional intermediate step of aligning the tilt axis with respect to the *Y* axis^5^. One of the novel contributions of this work is to provide a generalized mathematical formulation that avoids all these intermediate operations, explicitly taking into account the respective centers in the tilted and the untilted images, as well as generalizing the “stretching factor” to a “stretching matrix”, able to account for an arbitrary orientation of the tilt axis with respect to the image coordinate axis both in the tilted and untilted micrographs (Equation 12 of Supplementary Material). It is important to note that the use of a stretching operation relies on the assumption that the particle being reconstructed is infinitely thin (as rigorously proved in our Supplemental Material). In the limit, for a globular protein, the stretching of the tilted projection to match the untilted one may do more harm than good. In our implementation in Xmipp^8^,^9^ the user is asked whether she desires to perform this stretching or not.
• Calculation of 3D alignment parameters: If we are to produce a single 3D reconstruction out of multiple image pairs, we need to not only align the tilted projection to the untilted one, but also all the untilted projections among themselves, so that they are compatible with a single 3D structure. Implicitly, this requirement is usually handled by relying on the series of intermediate steps described in the previous section, considering that a common centered reference is used for all pairs. However, since we are aiming at avoiding all intermediate operations, we need to explicitly consider this common centering of all pairs. In Supplementary Material (Section 3.2), we show how to account for these inaccuracies in the selection of the untilted coordinates. The parameters needed for the 3D reconstruction normally involve the determination of 3 Euler angles and 2 in-plane shifts. We show how to calculate them as a function of an arbitrary tilt axis location, relating selection errors in the tilted and untilted projections. Additionally, we also consider the in-plane rotation needed to bring each untilted projection into the same orientation as the volume to be reconstructed. Finally, our derivation allows the determination of the 3D alignment parameters for an arbitrary orientation of the tilt axis, avoiding in this way the interpolation needed by the traditional approach in which the tilt axis is assumed to be along the vertical image axis.
• Improving the estimation of in-plane alignment parameters: One of the problems of aligning tilted and untilted projections, as well as untilted projections among themselves, is the low SNR of the images. This problem can be alleviated if at least one of the images has a larger SNR. This is actually the case if instead of aligning with respect to experimental untilted projections, we align to the class average of the untilted projections. The class average has a larger SNR and makes the estimation of the alignment parameters more reliable. As a drawback, we need to account for a new geometrical transformation: the alignment between the experimental untilted projections and their corresponding class average. Section 3.3 in Supplementary Material shows how to incorporate this extra alignment into the 3D reconstruction parameters to be used with the tilted projections.
•   Inclusion of mirror images: Depending on the molecule, target resolution and experimental SNR, some of the untilted projections may be classified by the 2D classification algorithm into the same class by applying a mirroring operation (that is, images assigned to a class may correspond to either mirrored or unmirrored projections). In our experience, mirror images may account for between 10% and 20% of the images in a class. The standard RCT approach does not consider the mirroring effect, so it precludes the possibility of utilizing this extra 10–20% of images. In Supplementary Material (Section 5), we show how mirrors can be explicitly modelled, so that mirrored and unmirrored images can be jointly used in a single 3D reconstruction.
•  Revisited RCT procedure: All these changes result in a new RCT procedure that is summarized in Section 4 of Supplementary Material. It basically follows the same steps as in the standard RCT procedure, but it provides new formulas for the calculation of the 3D alignment parameters of the tilted projections (the main output of the RCT process) without intermediate operations, like setting the tilt axis vertically.

In summary, previously published RCT procedures had a number of geometrical simplifications that required the use of a number of intermediate steps. The consequence was the introduction of a number of degradation steps in the process, besides making the whole approach more error prone and difficult to automatize and trace. In fact, it is probably the higher chance to make errors in these intermediate steps the main drawback of traditional approaches to RCT. In our approach we have relaxed these simplifications, providing general formulas for an arbitrary orientation of the tilt axis and arbitrary large errors in picking positions. We have also included the possibility of considering mirrored images and the alignment of the tilted images using a class average (which increases the SNR of one of the images being aligned and, consequently, has a higher alignment accuracy). Note that this methodology is compatible with the posterior alignment of all reconstructed volumes (from different 2D classes and corresponding RCT reconstructions) into a single volume in which the missing cone is complemented by information from other volumes^7^. However, this alignment has to be performed with care because the different volumes may correspond to different conformations. In this case, an alignment and classification approach may be preferred^10^. The improvements brought in by our geometrical framework goes well beyond interpolation errors or slight resolution improvements. The possible accumulation of geometrical errors in the process, from which the traditional RCT theory was more susceptible to suffer, may result in 3D reconstructions which can be greatly improved, as shown by our results. Additionally, by omitting intermediate steps, we make the whole RCT workflow to be easily traceable, improving the reporting and reproducibiity of the results.

## Results

To show a practical case of the use of these new geometrical relationships into a reconstruction procedure, we present here the results obtained with C3b as a test sample. This protein is one of the members of the complement system, a major component of innate immunity that comprises over thirty soluble and membrane-associated proteins having crucial roles in pathogen and apoptotic clearance, immune complexes handling, and modulation of adaptive immune responses. The central event in the complement activation cascade is the proteolytic cleavage of C3 to generate the activated fragment C3b. When C3b is generated, a reactive thioester group is exposed resulting in covalent attachment of C3b to pathogenic or apoptotic surfaces and thereby inducing several biological processes^11^,^12^.

**Figure 1:**
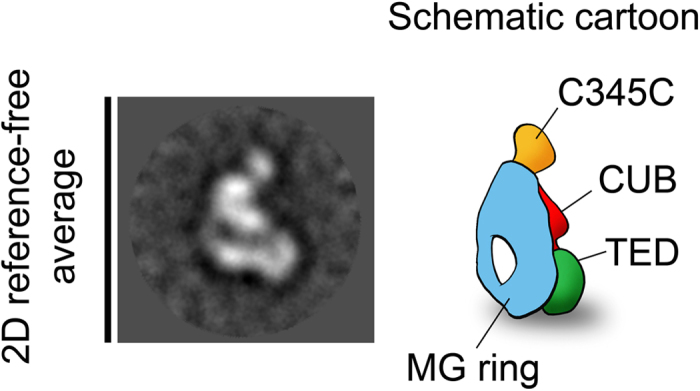
Selected 2D reference-free average showing a typical view of C3b. The cartoon representation highlights the location of the MG ring and C345C and TED domains.

C3b is a small multidomain molecule (180 kDa), which poses a challenge to the accuracy of orientation determination of individual single particles, since this process depends critically on molecular mass^13^. However, we wished to present a representative case of the broad range of relatively small and probably labile complexes that can now be studied at high resolution by cryo-EM. C3b images were subjected to 2D reference-free classification to group those images coming from similar views of the protein, revealing several detailed structural features. C3b appeared on the EM support film in different orientations, but the most common view showed the macroglobulin (MG) ring clearly visible with a hole at the bottom end of the ring as well as globular densities corresponding to C345C (complement protein subcomponents C1r/C1s, urchin embryonic growth factor and bone morphogenetic protein 1) and TED (thioester containing domain) domains at the top and at the lower part of the ring, respectively (see Fig. 1).

A few *μ*l of freshly purified complexes were adsorbed onto glow-discharged carbon-coated grids and negatively stained with 2% (mass/vol) uranyl formate (for a description of the purification process the reader is referred to^14^). Grids were visualized in a JEOL 1230 transmission electron microscope operated at 100 kV and a TemCam-F416 detector from Tietz Video and Image Processing Systems (TVIPS) using EM-TOOLS (TVIPS). Micrographs were recorded under low-dose conditions at a nominal magnification of 39,499x. Untilted (0°) and tilted (45°) micrographs were sequentially taken from a particular area in the grid following an RCT data collection scheme. Three thousands pairs of untilted and tilted projections were manually selected using Xmipp^8^,^9^.

An independent data set was obtained to serve as control for the RCT reconstruction. The collection of non-tilted micrographs was complemented by additional micrographs recorded at 30° and 45° tilting in order to increase the number of views obtained for C3b. A total of 32,595 C3b images were manually selected using e2boxer.py^15^ and subjected to 2D reference-free alignment and classification using Xmipp^16^. An initial volume was obtained using e2initialmodel.py from eman2. To investigate the presence of conformational heterogeneity, the data was refined using the 3D maximum-likelihood classification scheme implemented in RELION^17^. After convergence of the 3D classification refinement, the dataset was sorted into different homogeneous subsets (details of this analysis are given in^14^ and^18^). The output volume of the most abundant conformation was further refined using the 3D-autorefine-procedure implemented in RELION and the projection matching refinement procedure implemented in Xmipp^19^. The resolution of the structure was estimated as 26Å using Fourier shell correlation of two independent reconstructions and a cutoff of 0.5. This 3D map will be referred to in the following as the control map. Note that we intentionally used a completely different image processing workflow to serve as control, since the use of RCT as starting volume for a 3D reconstruction would invalidate the comparison between the RCT reconstruction and the final result. The biological interpretation of the structure of C3b is reported in^14^ and^18^.

**Figure 2:**
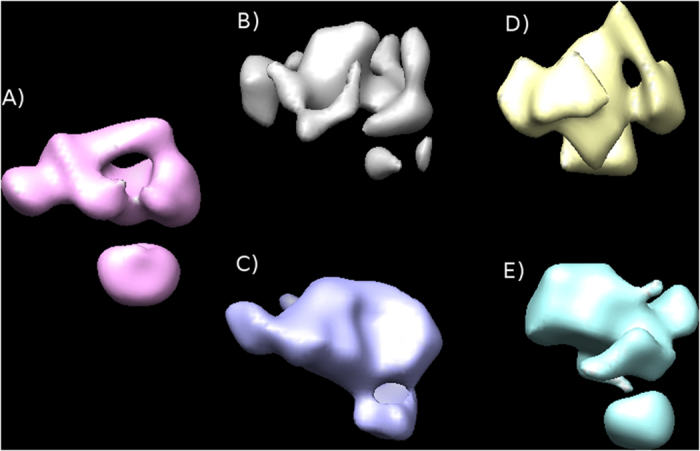
(**A**) Control map of C3b refined from 32,595 images. RCT applied to small 2D classes (less than 20 image pairs): (**B**) traditional RCT workflow as implemented in Spider (20 image pairs have been used); (**C**) new RCT workflow as implemented in XMIPP (13 image pairs used). RCT applied to medium size classes (about 60-80 image pairs): (**D**) traditional RCT workflow as implemented in Spider (79 image pairs used); (**E**) new RCT workflow as implemented in XMIPP (61 image pairs used).

We then compared the reconstructions obtained following both the new RCT procedure (as implemented in Xmipp) and the traditional RCT workflow (as described in^20^ and carefully detailed in http://spider.wadsworth.org/spider_doc/spider/docs/techs/ranconical/docs/rct.htm, which also performs a certain 2D classification), to the control map of C3b. In RCT, it is normally recommended to have small, very homogeneous 2D classes of untilted images, perform their 3D reconstruction using the RCT methodology, and then combine the different 3D structures to reduce the missing cone. We have followed the same strategy and have selected two examples of 2D classes with low and medium number of images (less than 20, and about 60–80). As can be appreciated in Fig. 2, the volumes reconstructed using the new RCT workflow are more similar to the control map than those obtained using the traditional RCT approach. The similarity is even more striking when we analyze the central slices of the aligned volumes (Supplementary Figs 1, 2, 3, 4 and 5) (it should be mentioned that this comparison can only address the gross morphology of the reconstructions, among other reasons because the sample is heterogeneous and the particular classes used in each analysis may not fully correspond to the same structure).

## Discussion

The importance of RCT in the analysis of macromolecular complexes by Electron Microscopy is unquestionable. It provides a reference-free initial map that can be used for subsequent refinement using an independent set of images. To date, it has successfully performed its task, contributing to many important biomedical results. However, some simplifications underlying its mathematical derivation required a number of intermediate, otherwise unnecessary, operations, which degraded the information, increased the probability of errors and difficulted a complete tracing of the processing workflow. Considering the new capabilities of cryo-EM thanks to the direct detectors and the large range of new biomedical systems it is expected to be applied to, it is of paramount importance to ensure that RCT can be routinely applied to its fullest extent. In an effort to streamline the mathematical formulation of RCT, we provide in this work a generalized derivation of the relationships between the untilted and tilted projections along with the class average of the untilted projections. This generalization includes the user picking errors as part of the model and, consequently, it is capable of correcting them, resulting in a better 3D reconstruction. It also shows how to directly align the tilted images to the class average of the untilted projections. In this way, the direct estimation of the in-plane shifts becomes much more reliable and accurate since one of the images being compared (the class average) has a much larger SNR. Additionally, it allows the inclusion of mirrored projections, thus increasing the number of images that can be used during the reconstruction. Finally, our mathematical derivation no longer requires the tilt axis to be aligned with the vertical axis, avoiding in this way interpolation errors. The use of this generalized formulation results in a simpler method, with less chances of user errors and potential intermediate degradations. As a practical case, we show how the implementation of this new approach in Xmipp can indeed obtain very meaningful RCT reconstructions with a very low number of images (13 in our example), requiring less steps than traditional approaches. This possibility opens new perspectives in the treatment of RCT reconstructions as if they were subtomograms, making use of all new alignment and classification procedures that are currently being developed in the field.

## Additional Information

**How to cite this article**: Sorzano, C.O.S. *et al.* Cryo-EM and the elucidation of new macromolecular structures: Random Conical Tilt revisited. *Sci. Rep.* **5**, 14290; doi: 10.1038/srep14290 (2015).

## Supplementary Material

Supplementary Material
