# Supplementary material for: Cryo-EM and the elucidation of new macromolecular structures: Random Conical Tilt revisited
Source: Sci Rep. 2015 Sep 22;5:14290. doi: 10.1038/srep14290 (PMC4585738; doi:10.1038/srep14290)
Supplement: Supplementary Material [file srep14290-s1.pdf]

Supplementary Material: Cryo-EM and the elucidation of new macromolecular structures: Random Conical Tilt revisited

C.O.S. Sorzano, M. Alcorlo, J.M. de la Rosa-Trevín, R. Melero, I. Foche, A. Zaldívar-Peraza, L. del Cano, J. Vargas, V. Abrishami, J. Otón, R. Marabini & J.M. Carazo

June 23, 2015

1 Determination of the tilt axis

Let us assume that each micrograph in the tilt pair is a projection of a large density volume containing all particles. Let us refer to this large volume as $V_{\text{micrograph}}(\tilde{\mathbf{r}}) : \mathbb{R}^3 \times \{1\} \rightarrow \mathbb{R}$ (note that the volume argument has been expressed in homogeneous coordinates, i.e., $\tilde{\mathbf{r}} = (x, y, z, 1)^T$ and T represents the transpose operator). We also express images in homogeneous coordinates. For instance, $I_{\text{micrograph},u}(\tilde{\mathbf{s}}) : \mathbb{R}^2 \times \{1\} \rightarrow \mathbb{R}$ represents the untilted projection image from the previous volume ($\tilde{\mathbf{s}} = (x, y, 1)^T$). The untilted micrograph can be modelled as

$$I_{\text{micrograph},u}(\tilde{\mathbf{s}}) = \int_{-\infty}^{\infty} V_{\text{micrograph}}(\tilde{R}_Z^{-1}(\alpha_u)\tilde{H}^T\tilde{\mathbf{s}})dz, \tag{1}$$

where

$$\tilde{H}^T = \begin{pmatrix} 1 & 0 & 0 \\ 0 & 1 & 0 \\ 0 & 0 & z \\ 0 & 0 & 1 \end{pmatrix}, \tag{2}$$

and $\tilde{R}_Z(\alpha_u)$ is the homogeneous rotation matrix about the Z axis by α_u degrees: $\tilde{R}_Z(\alpha_u) = \begin{pmatrix} \cos\alpha_u & \sin\alpha_u & 0 & 0 \\ -\sin\alpha_u & \cos\alpha_u & 0 & 0 \\ 0 & 0 & 1 & 0 \\ 0 & 0 & 0 & 1 \end{pmatrix}$. The rotation by α_u simply states that the tilt axis in the untilted image forms an angle α_u around the vertical axis. Similarly, we can model the tilted micrograph as

$$I_{\text{micrograph},t}(\tilde{\mathbf{s}}) = \int_{-\infty}^{\infty} V_{\text{micrograph}}((\tilde{T}\tilde{R}_Z(\alpha_t)\tilde{R}_Y(\theta))^{-1}\tilde{H}^T\tilde{\mathbf{s}})dz, \tag{3}$$

where θ is the tilt angle, $\tilde{R}_Y(\theta) = \begin{pmatrix} \cos\theta & 0 & -\sin\theta & 0 \\ 0 & 1 & 0 & 0 \\ \sin\theta & 0 & \cos\theta & 0 \\ 0 & 0 & 0 & 1 \end{pmatrix}$ is the rotation matrix around Y, and $\tilde{T} = \begin{pmatrix} 1 & 0 & 0 & t_{\mathrm{micrograph},X} \\ 0 & 1 & 0 & t_{\mathrm{micrograph},Y} \\ 0 & 0 & 1 & 0 \\ 0 & 0 & 0 & 1 \end{pmatrix}$ represents a possible shift introduced when tilting the sample holder. In a way, the formulation above can be seen as an extension of the work in [Guckenberger(1982)].

Eqs. (1) and (3) can be used to relate coordinates in the 3D volume $V_{\mathrm{micrograph}}$ to their projection locations. Particularly, consider the 3D homogeneous location $\tilde{\mathbf{r}}$, that projects into the untilted and tilted micrographs in locations

$$\begin{array}{l} \tilde{\mathbf{s}}_u = \tilde{H}_0 \tilde{R}_Z(\alpha_u)\tilde{\mathbf{r}} \\ \tilde{\mathbf{s}}_t = \tilde{H}_0 \tilde{T} \tilde{R}_Z(\alpha_t)\tilde{R}_Y(\theta)\tilde{\mathbf{r}} \end{array} \tag{4}$$

where $\tilde{H}_0$ is the $\tilde{H}$ matrix in Eq. (2) with $z = 0$. If we now restrict ourselves to 3D locations in the XY plane (i.e., $\tilde{\mathbf{r}} = (x, y, 0, 1)^T$), then we can recover $\tilde{\mathbf{r}}$ from its location in the untilted micrograph as $\tilde{\mathbf{r}} = \tilde{R}_Z^{-1}(\alpha_u)\tilde{H}_0^T \tilde{\mathbf{s}}_u$. Substituting in the expression for $\tilde{\mathbf{s}}_t$, we get

$$\tilde{\mathbf{s}}_t = \tilde{H}_0 \tilde{T} \tilde{R}_Z(\alpha_t)\tilde{R}_Y(\theta)\tilde{R}_Z^{-1}(\alpha_u)\tilde{H}_0^T \tilde{\mathbf{s}}_u \tag{5}$$

Similar equations without justification are presented by [Voss et al.(2009), Hauer et al.(2013)]. [Shatsky et al.(2014)] presents an evolution of this formulation in which particles are considered to be in a slightly bent grid.

If we know corresponding pairs of landmark locations in the untilted and tilted micrographs, this equation allows us to estimate the orientation of the tilt axis in both micrographs. From the knowledge of corresponding landmarks we can compute a matrix that meets in a least-squares sense that

$$\tilde{\mathbf{s}}_t = \tilde{A}\tilde{\mathbf{s}}_u = \begin{pmatrix} a_{11} & a_{12} & a_{13} \\ a_{21} & a_{22} & a_{23} \\ 0 & 0 & 1 \end{pmatrix} \tilde{\mathbf{s}}_u. \tag{6}$$

a_{13} and a_{23} directly provide an estimate for $\tilde{T}$. Then, we need to find angles α_u, α_t and θ such that the 2×2 top-left submatrix of $\tilde{E} = \tilde{R}_Z(\alpha_t)\tilde{R}_Y(\theta)\tilde{R}_Z^{-1}(\alpha_u)$ approximates as well as possible the 2×2 top-left submatrix of A. In fact, Eq. (36) in [Radermacher(1988)] provides an excellent estimate of θ, and [Voss et al.(2009)] introduces a weighted version of this estimate. α_u and α_t can be determined by any optimization algorithm that minimizes the error between the 2×2 top-left submatrix of $\tilde{A}$ and the 2×2 top-left submatrix of $\tilde{E}$.

This derivation is totally equivalent to the one presented by Dr. Radermacher [Radermacher(1988)] and already used in the field [Hegerl et al.(1991)]. However, the formulation has been presented in a different way to highlight an

important limitation. Indeed, the determination of the tilt axis is imperfect due to the assumption that image landmarks correspond to 3D points in the XY plane (i.e., $\tilde{\mathbf{r}} = (x, y, 0, 1)^T$). This does not need to be necessarily the case, since each particle may have a different height within the sample holder. Although it is feasible to further refine the estimate of the tilt axis location to include the different height effect, this is not normally done in the field, and it will not be done in this work. For thin samples (as is normally the case in EM) and due to error cancellation effects, we do not expect this error to be of a large magnitude.

2 Image formation model without mirrors

Let $V_{\text{ref}}(\tilde{\mathbf{r}}) : \mathbb{R}^3 \times \{1\} \to \mathbb{R}$ be a density volume (note that the volume argument has been expressed in homogeneous coordinates, i.e., $\mathbf{r} = (x, y, z, 1)^T$, T represents the transpose operator). This is the volume we would like to recover using RCT. The micrograph volume in the previous section, $V_{\text{micrograph}}(\tilde{\mathbf{r}})$, is supposed to be composed of multiple copies of this $V_{\text{ref}}(\tilde{\mathbf{r}})$ in different orientations and spatial locations.

Picking the particles in the tilt pair amounts to identifying the locations of the different copies of $V_{\text{ref}}(\tilde{\mathbf{r}})$ in the untilted and tilted micrographs. We will refer to the extracted particles from the untilted micrograph as the untilted images. 2D classification of the untilted images amounts to sorting the images according to the different orientations of $V_{\text{ref}}(\tilde{\mathbf{r}})$.

Once a single 2D class is selected, we have chosen a particular orientation of $V_{\text{ref}}(\tilde{\mathbf{r}})$ (with no loss of generality we will assume that $V_{\text{ref}}(\tilde{\mathbf{r}})$ corresponds to the particular orientation selected by the 2D class) and its 0° projection should be similar to the 2D class average calculated by the 2D classification algorithm ($I_{\text{ref},0^\circ}(\tilde{\mathbf{s}}) \approx I_u^{\text{2Dclass}}(\tilde{\mathbf{s}})$). Note that we do not have experimental access to $I_{\text{ref},0^\circ}$ since it is a projection calculated from a volume we do not actually have. However, we do have access to I_u^{2Dclass} since it is the result of averaging all experimental images assigned to the same 2D class by the classifier.

Each image pair corresponds to the projection of a certain copy of $V_{\text{ref}}(\tilde{\mathbf{r}})$, that we will refer to as $V(\tilde{\mathbf{r}})$. The observed untilted image corresponds to a 0° projection of this volume

$$I_u(\tilde{\mathbf{s}}) = \int_{-\infty}^{\infty} V(\tilde{R}_Z^{-1}(\alpha_u)\tilde{H}^T\tilde{\mathbf{s}})dz, \tag{7}$$

where we have explicitly accounted for the fact that the tilt axis in the untilted micrograph is not aligned with the vertical axis. The corresponding tilted image would be

$$I_t(\tilde{\mathbf{s}}) = \int_{-\infty}^{\infty} V((\tilde{R}_Z(\alpha_t)\tilde{R}_Y(\theta))^{-1}\tilde{H}^T\tilde{\mathbf{s}})dz. \tag{8}$$

However, the user seldom picks exactly at corresponding points between the untilted and tilted images and, therefore, a small shift can be expected. The

actual tilted image selected by the user would then correspond to

$$I_t(\tilde{\mathbf{s}}) = \int_{-\infty}^{\infty} V((\tilde{R}_Z(\alpha_t)\tilde{R}_Y(\theta))^{-1}\tilde{H}^T\tilde{T}_t^{-1}\tilde{\mathbf{s}})dz, \quad (9)$$

being $\tilde{T}_t$ a 2D homogeneous matrix that reflects a shift introduced by the user picking.

3 Alignment of the tilted image

Traditionally, the alignment has been done by comparing the untilted image and the corresponding stretched tilted image once they have been both corrected to align the tilt axis with the vertical axis [Zampighi et al.(2004), Zheng et al.(2007)] (in fact, Dr. Radermacher[Radermacher(1988)] suggests to do the stretching in the direction perpendicular to the tilt axis in the tilted image, but it does not give any specific formula to do it). However, both images have very low Signal-to-Noise Ratio (SNR) making the alignment quite difficult. Alternatively, it is possible to substitute the untilted image by the class average that has a much better SNR. In the following sections, we show how to perform this alignment. We divide the derivation in three parts: first, we present the formal definition of streching in the direction perpendicular to the tilt axis and present the assumptions that justifies the use of that stretching; second, we relate the orientation of the individual copies of the reconstructed volume to the standard position defined by the 2D class average; finally, we extend the traditional in-plane alignment method so that we can use the 2D class average.

3.1 Stretching the tilted image and its assumptions

Let us define a new volume in which the angle of the tilt axis in the untilted image has been corrected:

$$V'(\tilde{\mathbf{r}}) = V(\tilde{R}_Z^{-1}(\alpha_u)\tilde{\mathbf{r}}) \leftrightarrow V(\tilde{\mathbf{r}}) = V'(\tilde{R}_Z(\alpha_u)\tilde{\mathbf{r}}). \quad (10)$$

The experimental projections in terms of the new volume become:

$$\begin{aligned} I_u(\tilde{\mathbf{s}}) &= \int_{-\infty}^{\infty} V'(\tilde{H}^T\tilde{\mathbf{s}})dz \\ I_t(\tilde{\mathbf{s}}) &= \int_{-\infty}^{\infty} V'(\tilde{R}_Z(\alpha_u)(\tilde{R}_Z(\alpha_t)\tilde{R}_Y(\theta))^{-1}\tilde{H}^T\tilde{T}_t^{-1}\tilde{\mathbf{s}})dz = \int_{-\infty}^{\infty} V'(\tilde{E}^{-1}\tilde{H}^T\tilde{T}_t^{-1}\tilde{\mathbf{s}})dz, \end{aligned} \quad (11)$$

where $\tilde{E} = \tilde{R}_Z(\alpha_t)\tilde{R}_Y(\theta)\tilde{R}_Z(-\alpha_u)$. If V is infinitely thin and concentrated in the XY plane (that is $V(\tilde{\mathbf{r}}) = V(\tilde{\mathbf{r}}_X, \tilde{\mathbf{r}}_Y, 0)\delta(\tilde{\mathbf{r}}_Z)$, being $\delta(x)$ the Dirac's delta distribution), then so is V' (since it is only a Z-rotated version of V). In this

way, we have (see Appendix for its proof)

$$\begin{aligned} I_u(\tilde{\mathbf{s}}) &= V'(\tilde{H}_0^T\tilde{\mathbf{s}}) \\ I_t(\tilde{\mathbf{s}}) &= \int_{-\infty}^{\infty} V'(\tilde{E}^{-1}\tilde{H}^T\tilde{T}_t^{-1}\tilde{\mathbf{s}})dz = V'(\tilde{H}_0^T(\tilde{H}_0\tilde{E}\tilde{H}_0^T)^{-1}\tilde{T}_t^{-1}\tilde{\mathbf{s}}) \\ &= I_u((\tilde{H}_0\tilde{E}\tilde{H}_0^T)^{-1}\tilde{T}_t^{-1}\tilde{\mathbf{s}}) \end{aligned} \tag{12}$$

In the particular case that $\alpha_u = \alpha_t = 0$ (i.e., the tilt is performed around the Y axis in both images), then the previous equation becomes

$$\begin{array}{l} I_u(\tilde{\mathbf{s}}) = V'(\tilde{H}_0^T\tilde{\mathbf{s}}) \\ I_t(\tilde{\mathbf{s}}) = V'(\tilde{H}_0^T S\tilde{T}_t^{-1}\tilde{\mathbf{s}}) = I_u(S\tilde{T}_t^{-1}\tilde{\mathbf{s}}) \end{array} \tag{13}$$

where

$$S = \begin{pmatrix} \frac{1}{\cos(\theta)} & 0 & 0 \\ 0 & 1 & 0 \\ 0 & 0 & 1 \end{pmatrix}. \tag{14}$$

That is, the tilted image is a shifted and stretched (by a factor $\frac{1}{\cos(\theta)}$) version of the untilted one, as stated by Dr. Radermacher[Radermacher(1988)]. We, therefore, see that the traditional stretching for aligning RCT tilt pairs is a paticular case of the more general case given by Eq. (12), and more importantly, this derivation is only valid for infinitely thin objects. As soon as we depart from this assumption, the equality becomes only an approximation or may be even invalid for thick objects.

3.2 3D alignment parameters

The in-plane orientation of volume V' is not the same as the orientation of V_{ref} (in fact, this is the key of the success of Random Conical Tilt). Let us define $\tilde{M}_u$ as an affine matrix that encodes an in-plane rotation ($\tilde{R}_Z(\alpha'_u)$) followed by a translation ($\tilde{T}_u$), that is

$$\tilde{M}_u = \tilde{T}_u\tilde{R}_Z(\alpha'_u), \tag{15}$$

such that the two volumes are registered to each other, i.e.,

$$V_{\text{ref}}(\tilde{\mathbf{r}}) = V'(\tilde{M}_u^{-1}\tilde{\mathbf{r}}) \leftrightarrow V'(\tilde{\mathbf{r}}) = V_{\text{ref}}(\tilde{M}_u\tilde{\mathbf{r}}). \tag{16}$$

$\tilde{M}_u$ can be easily estimated from the transformation needed to align $I_u(\tilde{\mathbf{s}})$ to the 2D class average selected ($I_u^{\text{2Dclass}}(\tilde{\mathbf{s}})$) because

$$I_u^{\text{2Dclass}}(\tilde{\mathbf{s}}) \approx I_u((\tilde{H}_0\tilde{M}_u\tilde{H}_0^T)^{-1}\tilde{\mathbf{s}}). \tag{17}$$

Note that $\tilde{H}_0\tilde{M}_u\tilde{H}_0^T$ is a compact way of selecting the top-left 2×2 matrix of $\tilde{M}_u$ and making it a 2D homogeneous matrix.

Combining all this information, we can relate the experimentally observed images to the volume we aim to reconstruct by

$$\begin{aligned}
I_u(\tilde{\mathbf{s}}) &= \int\limits_{-\infty}^{\infty} V_{\mathrm{ref}}(\tilde{M}_u \tilde{H}^T \tilde{\mathbf{s}})dz \\
I_t(\tilde{\mathbf{s}}) &= \int\limits_{-\infty}^{\infty} V_{\mathrm{ref}}(\tilde{M}_u \tilde{R}_Z(\alpha_u)(\tilde{R}_Z(\alpha_t)\tilde{R}_Y(\theta))^{-1}\tilde{H}^T \tilde{T}_t^{-1}\tilde{\mathbf{s}})dz \\
&= \int\limits_{-\infty}^{\infty} V_{\mathrm{ref}}(\tilde{T}_u \tilde{R}_Z(\alpha_u')\tilde{R}_Z(\alpha_u)(\tilde{R}_Z(\alpha_t)\tilde{R}_Y(\theta))^{-1}\tilde{H}^T \tilde{T}_t^{-1}\tilde{\mathbf{s}})dz \qquad (18) \\
&= \int\limits_{-\infty}^{\infty} V_{\mathrm{ref}}(\tilde{T}_u (\tilde{R}_Z(\alpha_t)\tilde{R}_Y(\theta)\tilde{R}_Z(-(\alpha_u'+\alpha_u)))^{-1}\tilde{H}^T \tilde{T}_t^{-1}\tilde{\mathbf{s}})dz \\
&= \int\limits_{-\infty}^{\infty} V_{\mathrm{ref}}(\tilde{T}_u \tilde{E}^{-1}\tilde{H}^T \tilde{T}_t^{-1}\tilde{\mathbf{s}})dz,
\end{aligned}$$

where we have defined the matrix $\tilde{E}$ as $\tilde{E} = \tilde{R}_Z(\alpha_t)\tilde{R}_Y(\theta)\tilde{R}_Z(-(\alpha_u'+\alpha_u))$. This is an Euler rotation matrix in the convention ZYZ whose first rotation is around Z by $-(\alpha_u'+\alpha_u)$ degrees (rotational angle), the second is arout Y by θ degrees (tilt angle), and finally arout Z by α_t degrees (in-plane rotation).

In Xmipp [Sorzano et al.(2004)], the orientation and translation of a projection with respect to a volume are defined through the following relationship

$$I_t(\tilde{\mathbf{s}}) = \int\limits_{-\infty}^{\infty} V_{\mathrm{ref}}(\tilde{E}^{-1}\tilde{H}^T \tilde{T}_t'^{-1}\tilde{\mathbf{s}})dz. \qquad (19)$$

So we need to transform Eq. (18) into the form of Eq. (19). For doing so we find a new matrix $\tilde{T}_u'$ such that

$$I_t(\tilde{\mathbf{s}}) = \int\limits_{-\infty}^{\infty} V_{\mathrm{ref}}(\tilde{T}_u \tilde{E}^{-1}\tilde{H}^T \tilde{T}_t^{-1}\tilde{\mathbf{s}})dz = \int\limits_{-\infty}^{\infty} V_{\mathrm{ref}}(\tilde{E}^{-1}\tilde{T}_u'\tilde{H}^T \tilde{T}_t^{-1}\tilde{\mathbf{s}})dz \qquad (20)$$

The new $\tilde{T}_u'$ must, therefore, satisfy $\tilde{T}_u\tilde{E}^{-1} = \tilde{E}^{-1}\tilde{T}_u'$. It can be easily shown that

$$\tilde{T}_u' = \tilde{E}\tilde{T}_u\tilde{E}^{-1} \qquad (21)$$

is the translation matrix sought. By a change of variable, it can be easily proved that

$$I_t(\tilde{\mathbf{s}}) = \int\limits_{-\infty}^{\infty} V_{\mathrm{ref}}(\tilde{E}^{-1}\tilde{T}_u'\tilde{H}^T \tilde{T}_t^{-1}\tilde{\mathbf{s}})dz = \int\limits_{-\infty}^{\infty} V_{\mathrm{ref}}(\tilde{E}^{-1}\tilde{H}^T \tilde{T}_{u,2D}'\tilde{T}_t^{-1}\tilde{\mathbf{s}})dz, \qquad (22)$$

where $\tilde{T}_{u,2D}' = \tilde{H}_0\tilde{T}_u'\tilde{H}_0^T$ (this is a compact way of turning a 3D homogeneous translation matrix into a 2D homogeneous matrix by simply dropping the translation along Z).

Finally, we find that the translation needed by Eq. (19) is

$$\tilde{T}_t' = \tilde{T}_t\left(\tilde{T}_{u,2D}'\right)^{-1} \qquad (23)$$

3.3 Aligning the tilted images to the 2D class average

The derivation in the previous section allows us to align the untilted and tilted images in a tilt pair. However, these two images have very low SNR since both of them are experimental. We may substitute the untilted image by the class average produced by the classification algorithm. This class average has a larger SNR, and it is supposed to be identical to the untilted image except for some misalignment provided by Eq. (17). Let us now derive the relationship between $I_t(\tilde{\mathbf{s}})$ and $I_u^{\text{2Dclass}}(\tilde{\mathbf{s}})$ in the case of an infinitely thin sample (so that we can reuse the stretching theory above).

We reproduce here the main results of Eqs. (17) and (12)

$$\begin{aligned} I_t(\tilde{\mathbf{s}}) &= I_u((\tilde{E}^{2D})^{-1}\tilde{T}_t^{-1}\tilde{\mathbf{s}}) \\ I_u^{\text{2Dclass}}(\tilde{\mathbf{s}}) &\approx I_u((\tilde{M}_u^{2D})^{-1}\tilde{\mathbf{s}}) \end{aligned} \tag{24}$$

In which we have defined $\tilde{E}^{2D} = \tilde{H}_0\tilde{E}\tilde{H}_0^T$ and $\tilde{M}_u^{2D} = \tilde{H}_0\tilde{M}_u\tilde{H}_0^T$. From the first equation we can conclude that

$$I_u(\tilde{\mathbf{s}}) = I_t(\tilde{T}_t\tilde{E}^{2D}\tilde{\mathbf{s}}). \tag{25}$$

Therefore,

$$I_u^{\text{2Dclass}}(\tilde{\mathbf{s}}) \approx I_t(\tilde{T}_t\tilde{E}^{2D}(\tilde{M}_u^{2D})^{-1}\tilde{\mathbf{s}}). \tag{26}$$

From a computational point of view and for reasons that will become apparent now, it is more convenient to define two new auxiliary matrices:

$$\begin{aligned} \tilde{A}^{\text{2Dclass}} &= \tilde{M}_u^{2D}(\tilde{E}^{2D})^{-1} \\ \tilde{T}_t^{\text{2Dclass}} &= \tilde{A}^{\text{2Dclass}}\tilde{T}_t^{-1}(\tilde{A}^{\text{2Dclass}})^{-1} \end{aligned} \tag{27}$$

such that

$$I_u^{\text{2Dclass}}(\tilde{\mathbf{s}}) \approx I_t((\tilde{A}^{\text{2Dclass}})^{-1}(\tilde{T}_t^{\text{2Dclass}})^{-1}\tilde{\mathbf{s}}). \tag{28}$$

At this point, let us define the image

$$I_t^{\text{2Dclass}}(\tilde{\mathbf{s}}) = I_t((\tilde{A}^{\text{2Dclass}})^{-1}\tilde{\mathbf{s}}), \tag{29}$$

that is nothing more than the tilted image transformed so that it becomes in the same orientation as the 2D class average of the untilted images. Now we have

$$I_u^{\text{2Dclass}}(\tilde{\mathbf{s}}) \approx I_t^{\text{2Dclass}}((\tilde{T}_t^{\text{2Dclass}})^{-1}\tilde{\mathbf{s}}). \tag{30}$$

The translation encoded by $\tilde{T}_t^{\text{2Dclass}}$ can be found at this point by simple cross correlation in Fourier space, and then we recover the sought translation by

$$\tilde{T}_t = (\tilde{A}^{\text{2Dclass}})^{-1}(\tilde{T}_t^{\text{2Dclass}})^{-1}\tilde{A}^{\text{2Dclass}}. \tag{31}$$

4 Revisited RCT algorithm

According to the theory explained in this document, a RCT reconstruction can be performed as follows:

For each pair of micrographs (untilted and tilted):

- Step 1: Mark a set of corresponding particle centers (set of $\tilde{s}_u$ and $\tilde{s}_t$ coordinates).
- Step 2: Estimate by Least Squares or any other method a matrix that transforms untilted coordinates into tilted coordinates (see Eq. (6)).
- Step 3: Use Eq. (36) in [Radermacher(1988)] to estimate the tilt angle, θ.
- Step 4: Use any optimization algorithm to find the angles α_u and α_t that minimize the error between the 2×2 top-left submatrix of $\tilde{A}$ and the 2×2 top-left submatrix of $\tilde{E}$ (see Eq. (6) and text around).
- Step 5: Extract the particle image pairs $I_u(\tilde{\mathbf{s}})$ and $I_t(\tilde{\mathbf{s}})$.

For all untilted particle projections ($I_u(\tilde{\mathbf{s}})$):

- Step 6: Perform a 2D classification into different classes.
- Step 7: Choose a certain class for which the 3D model is desired, $I_u^{\mathrm{2Dclass}}(\tilde{\mathbf{s}})$

For all particle pairs ($I_u(\tilde{\mathbf{s}})$, $I_t(\tilde{\mathbf{s}})$):

- Step 8: Compute $\tilde{M}_u$ such that the 2D class and the untilted image become aligned (see Eq. (17))
- Step 9: If the particle to reconstruct is thin enough so that stretching the tilted image is not a strong violation, stretch the tilted image to look like the untilted 2D class average according to Eq. (29).
- Step 10: Find the best shift, $\tilde{T}_t^{\mathrm{2Dclass}}$, so that the image in Step 9 is registered with the 2D class (see Eq. (30)).
- Step 11: Estimate the particle picking misalignment according to Eq. (31).
- Step 12: Compute the shift needed by the reconstruction algorithm, in the case of Xmipp use Eq. (23).
- Step 13: The ZYZ Euler angles of the tilted image are $(-(\alpha'_u + \alpha_u), \theta, \alpha_t)$ (first, second, and third rotation, respectively).

Once all particle geometry parameters have been determined:

- Step 14: Use any 3D reconstruction algorithm using the tilted images (without stretching) and the shifts and Euler angles calculated in Steps 12 and 13.
- Step 15: Angles and shifts can be further refined using any projection matching algorithm.

5 Geometrical framework with mirrors

Let us now consider the case in which there are untilted images in the 2D class related by mirroring over the Y axis (X is flipped). This is the case of some classification algorithms as CL2D [Sorzano et al.(2010)] and ML2D [Scheres et al.(2005)]. The traditional approach to handling mirrors in RCT has been to discard those images that are mirrors or to classify them into a separate class. But doing so discards useful information that can be exploited to construct a more reliable RCT model.

Eqs. (7) and (9) are still valid, only that they now refer to a volume V_{mirror}, that is

$$\begin{array}{l} I_u(\tilde{\mathbf{s}}) = \int\limits_{-\infty}^{\infty} V_{\text{mirror}}(\tilde{R}_Z^{-1}(\alpha_u)\tilde{H}^T\tilde{\mathbf{s}})dz \\ I_t(\tilde{\mathbf{s}}) = \int\limits_{-\infty}^{\infty} V_{\text{mirror}}((\tilde{R}_Z(\alpha_t)\tilde{R}_Y(\theta))^{-1}\tilde{H}^T\tilde{T}_t^{-1}\tilde{\mathbf{s}})dz, \end{array} \tag{32}$$

with

$$V_{\text{mirror}}(\tilde{\mathbf{r}}) = V(\tilde{R}_Y^{-1}(\pi)\tilde{\mathbf{r}}). \tag{33}$$

Note that now Eqs. (15) and (16) become

$$\begin{array}{l} \tilde{M}_u = \tilde{R}_Y^{-1}(\pi)\tilde{T}_u\tilde{R}_Z(\alpha'_u) \\ V_{\text{ref}}(\tilde{\mathbf{r}}) = V'_{\text{mirror}}(\tilde{M}_u^{-1}\tilde{\mathbf{r}}) \leftrightarrow V'_{\text{mirror}}(\tilde{\mathbf{r}}) = V_{\text{ref}}(\tilde{M}_u\tilde{\mathbf{r}}), \end{array} \tag{34}$$

and Eq. (17) is still valid.

The relationship between the tilted image and V_{ref} (Eq. (18)) now is

$$\begin{array}{rcl} I_t(\tilde{\mathbf{s}}) & = & \int\limits_{-\infty}^{\infty} V_{\text{ref}}(\tilde{M}_u\tilde{R}_Z(\alpha_u)(\tilde{R}_Z(\alpha_t)\tilde{R}_Y(\theta))^{-1}\tilde{H}^T\tilde{T}_t^{-1}\tilde{\mathbf{s}})dz \\ & = & \int\limits_{-\infty}^{\infty} V_{\text{ref}}(\tilde{R}_Y^{-1}(\pi)\tilde{T}_u\tilde{R}_Z(\alpha'_u)\tilde{R}_Z(\alpha_u)\tilde{R}_Y^{-1}(\theta)\tilde{R}_Z^{-1}(\alpha_t)\tilde{H}^T\tilde{T}_t^{-1}\tilde{\mathbf{s}})dz \\ & = & \int\limits_{-\infty}^{\infty} V_{\text{ref}}(\tilde{R}_Y^{-1}(\pi)\tilde{T}_u\tilde{R}_Z(\alpha'_u+\alpha_u)\tilde{R}_Y(\theta)^{-1}\tilde{R}_Z^{-1}(\alpha_t)\tilde{H}^T\tilde{T}_t^{-1}\tilde{\mathbf{s}})dz \end{array} \tag{35}$$

Now, we define $\tilde{T}_{u,\text{mirror}} = \tilde{R}_Y(\theta+\pi)^{-1}\tilde{T}_u\tilde{R}_Y(\theta+\pi)$ (this simply amounts to change the sign of the shift in X). In this way, we can rewrite the previous expression as

$$I_t(\tilde{\mathbf{s}}) = \int\limits_{-\infty}^{\infty} V_{\text{ref}}(\tilde{T}_{u,\text{mirror}}\tilde{R}_Y^{-1}(\pi)\tilde{R}_Z(\alpha'_u+\alpha_u)\tilde{R}_Y(\theta)^{-1}\tilde{R}_Z^{-1}(\alpha_t)\tilde{H}^T\tilde{T}_t^{-1}\tilde{\mathbf{s}})dz \tag{36}$$

At this point we make use of the fact $\tilde{R}_Y^{-1}(\pi)\tilde{R}_Z(\alpha'_u+\alpha_u) = \tilde{R}_Z(-(\alpha'_u+\alpha_u))\tilde{R}_Y^{-1}(\pi)$, then

$$\begin{array}{rcl} I_t(\tilde{\mathbf{s}}) & = & \int\limits_{-\infty}^{\infty} V_{\text{ref}}(\tilde{T}_{u,\text{mirror}}\tilde{R}_Z(-(\alpha'_u+\alpha_u))\tilde{R}_Y(\theta+\pi)^{-1}\tilde{R}_Z^{-1}(\alpha_t)\tilde{H}^T\tilde{T}_t^{-1}\tilde{\mathbf{s}})dz \\ & = & \int\limits_{-\infty}^{\infty} V_{\text{ref}}(\tilde{T}_{u,\text{mirror}}\tilde{E}_{\text{mirror}}^{-1}\tilde{H}^T\tilde{T}_t^{-1}\tilde{\mathbf{s}})dz \end{array} \tag{37}$$

where $\tilde{E}_{\text{mirror}} = \tilde{R}_Z(\alpha_t)\tilde{R}_Y(\theta+\pi)\tilde{R}_Z(\alpha'_u+\alpha_u)$.

Regarding the tilt image alignment, we can rewrite Eq. (11) in terms of the mirrored volume

$$\begin{aligned} I_u(\tilde{\mathbf{s}}) &= \int_{-\infty}^{\infty} V'_{\text{mirror}}(\tilde{H}^T\tilde{\mathbf{s}})dz \\ I_t(\tilde{\mathbf{s}}) &= \int_{-\infty}^{\infty} V'_{\text{mirror}}(\tilde{R}_Z(\alpha_u)(\tilde{R}_Z(\alpha_t)\tilde{R}_Y(\theta))^{-1}\tilde{H}^T\tilde{T}_t^{-1}\tilde{\mathbf{s}})dz, \end{aligned} \tag{38}$$

with $V'_{\text{mirror}}(\tilde{\mathbf{r}}) = V'(\tilde{R}_Y^{-1}(\pi)\tilde{\mathbf{r}})$. Substituting V'_{mirror} in the previous expression, we get

$$\begin{aligned} I_u(\tilde{\mathbf{s}}) &= \int_{-\infty}^{\infty} V'(\tilde{R}_Y^{-1}(\pi)\tilde{H}^T\tilde{\mathbf{s}})dz \\ I_t(\tilde{\mathbf{s}}) &= \int_{-\infty}^{\infty} V'(\tilde{R}_Y^{-1}(\pi)\tilde{R}_Z(\alpha_u)\tilde{R}_Y^{-1}(\theta)\tilde{R}_Z^{-1}(\alpha_t)\tilde{H}^T\tilde{T}_t^{-1}\tilde{\mathbf{s}})dz \\ &= \int_{-\infty}^{\infty} V'(\tilde{R}_Z(-\alpha_u)\tilde{R}_Y^{-1}(\theta+\pi)\tilde{R}_Z^{-1}(\alpha_t)\tilde{H}^T\tilde{T}_t^{-1}\tilde{\mathbf{s}})dz, \\ &= \int_{-\infty}^{\infty} V'(\tilde{E}_{\text{mirror}}^{-1}\tilde{H}^T\tilde{T}_t^{-1}\tilde{\mathbf{s}})dz, \end{aligned} \tag{39}$$

where $\tilde{E}_{\text{mirror}} = \tilde{R}_Z(\alpha_t)\tilde{R}_Y(\theta+\pi)\tilde{R}_Z(\alpha_u)$. Again, as we did for Eq. (12), we can show (see Appendix) that

$$I_t(\tilde{\mathbf{s}}) \quad = \quad I_u(\tilde{M}(\tilde{H}_0\tilde{E}_{\text{mirror}}\tilde{H}_0^T)^{-1}\tilde{T}_t^{-1}\tilde{\mathbf{s}}), \tag{40}$$

where $\tilde{M} = \tilde{H}_0\tilde{R}_Y^{-1}(\pi)\tilde{H}_0^T$. Combining this equation with Eq. (17) we have

$$I_u^{\text{2Dclass}}(\tilde{\mathbf{s}}) \approx I_t(\tilde{T}_t\tilde{E}_{\text{mirror}}^{2D}\tilde{M}(\tilde{M}_u^{2D})^{-1}\tilde{\mathbf{s}}), \tag{41}$$

that is the mirror version equivalent of Eq. (26). From this point on, we can follow the same derivation as in Section 3.3 except that we need to define

$$\tilde{A}^{\text{2Dclass}} = \tilde{M}_u^{2D}\tilde{M}(\tilde{E}^{2D})^{-1} \tag{42}$$

Therefore, the only modifications to the previous RCT algorithm are:

- Step 8: Compute $\tilde{M}_u$ such that the 2D class and the untilted image become aligned (see Eq. (34))
- Step 9: If the particle to reconstruct is thin enough so that stretching the tilted image is not a strong violation, stretch the tilted image to look like the untilted 2D class average according to Eq. (29) (but using $\tilde{A}^{\text{2Dclass}}$ as defined in Eq. (42)).
- Step 11: Estimate the particle picking misalignment according to Eq. (31) (but using $\tilde{A}^{\text{2Dclass}}$ as defined in Eq. (42))
- Step 13: The ZYZ Euler angles of the tilted image are $(\alpha'_u+\alpha_u, \theta+\pi, \alpha_t)$ (first, second, and third rotation, respectively).

Appendix

Proof of Eq. (12)

Let us prove Eq. (12), which we reproduce here:

$$I_t(\tilde{\mathbf{s}}) = \int_{-\infty}^{\infty} V'(\tilde{E}^{-1}\tilde{H}^T\tilde{T}_t^{-1}\tilde{\mathbf{s}})dz = V'(\tilde{H}_0^T(\tilde{H}_0\tilde{E}\tilde{H}_0^T)^{-1}\tilde{T}_t^{-1}\tilde{\mathbf{s}}) \tag{43}$$

with $\tilde{E} = \tilde{R}_Z(\alpha_t)\tilde{R}_Y(\theta)\tilde{R}_Z(-\alpha_u)$. Note that $\tilde{H}^T\tilde{T}_t^{-1}\tilde{\mathbf{s}} = (\mathbf{s}_X - t_{t,X}, \mathbf{s}_Y - t_{t,Y}, z, 1)^T$. For notation simplicity, we define two new variables such that $\tilde{H}^T\tilde{T}_t^{-1}\tilde{\mathbf{s}} = (x, y, z, 1)^T$. With this notation the argument of V' within the integral simplifies to

$$\tilde{E}^{-1}\tilde{H}^T\tilde{T}_t^{-1}\tilde{\mathbf{s}} = \begin{pmatrix} x(\sin(\alpha_u)\sin(\alpha_t) + \cos(\alpha_u)\cos(\alpha_t)\cos(\theta)) + y(\sin(\alpha_u)\cos(\alpha_t) - \cos(\alpha_u)\sin(\alpha_t)\cos(\theta)) + z\cos(\alpha_u)\sin(\theta) \\ x(\cos(\alpha_u)\sin(\alpha_t) - \sin(\alpha_u)\cos(\alpha_t)\cos(\theta)) + y(\cos(\alpha_u)\cos(\alpha_t) + \sin(\alpha_u)\sin(\alpha_t)\cos(\theta)) - z\sin(\alpha_u)\sin(\theta) \\ -x\cos(\alpha_t)\sin(\theta) + y\sin(\alpha_t)\sin(\theta) + z\cos(\theta) \\ 1 \end{pmatrix}. \tag{44}$$

Since V' is a delta distribution concentrated in the XY-plane, its integral along Z coincides with the value of V' when the third coordinate is 0, that is

$$-x\cos(\alpha_t)\sin(\theta) + y\sin(\alpha_t)\sin(\theta) + z\cos(\theta) = 0,$$

or, what is the same, when

$$z = x\cos(\alpha_t)\tan(\theta) - y\sin(\alpha_t)\tan(\theta).$$

Substituting this value into Eqs. (44) and (43), we have

$$I_t(\tilde{\mathbf{s}}) = \int_{-\infty}^{\infty} V'(\tilde{E}^{-1}\tilde{H}^T\tilde{T}_t^{-1}\tilde{\mathbf{s}})dz = V'(\tilde{H}_0^T\tilde{E}'\tilde{T}_t^{-1}\tilde{\mathbf{s}}) \tag{45}$$

where

$$\tilde{E}' = \begin{pmatrix} \sin(\alpha_u)\sin(\alpha_t) + \frac{\cos(\alpha_u)\cos(\alpha_t)}{\cos(\theta)} & \sin(\alpha_u)\cos(\alpha_t) - \frac{\cos(\alpha_u)\sin(\alpha_t)}{\cos(\theta)} & 0 \\ \cos(\alpha_u)\sin(\alpha_t) - \frac{\sin(\alpha_u)\cos(\alpha_t)}{\cos(\theta)} & \cos(\alpha_u)\cos(\alpha_t) + \frac{\sin(\alpha_u)\sin(\alpha_t)}{\cos(\theta)} & 0 \\ 0 & 0 & 1 \end{pmatrix}. \tag{46}$$

It turns out that $\tilde{E}'$ is the homogeneous inverse matrix of the 2×2 top-left submatrix of $\tilde{E}$, i.e.

$$\tilde{E}' = (\tilde{H}_0\tilde{E}\tilde{H}_0^T)^{-1}. \tag{47}$$

Substituting $\tilde{E}'$ by its value in Eq. (45), we obtain Eq. (43), which finishes the proof.

Proof of Eq. (40)

Let us start with Eq. (39), which we reproduce here for convenience:

$$\begin{aligned} I_u(\tilde{\mathbf{s}}) &= \int_{-\infty}^{\infty} V'(\tilde{R}_Y^{-1}(\pi)\tilde{H}^T\tilde{\mathbf{s}})dz \\ I_t(\tilde{\mathbf{s}}) &= \int_{-\infty}^{\infty} V'(\tilde{E}_{\text{mirror}}^{-1}\tilde{H}^T\tilde{T}_t^{-1}\tilde{\mathbf{s}})dz, \end{aligned} \tag{48}$$

with $\tilde{E}_{\text{mirror}} = \tilde{R}_Z(\alpha_t)\tilde{R}_Y(\theta+\pi)\tilde{R}_Z(\alpha_u)$. Since V' corresponds to an infinitely thin volume, we know

$$I_u(\tilde{\mathbf{s}}) = V'(\tilde{R}_Y^{-1}(\pi)\tilde{H}_0^T\tilde{\mathbf{s}}) = V'(\tilde{H}_0^T\tilde{M}\tilde{\mathbf{s}}). \tag{49}$$

On the other hand, making the same change of variable as in the previous proof ($\tilde{H}^T\tilde{T}_t^{-1}\tilde{\mathbf{s}} = (x, y, z, 1)^T$)

$$\tilde{E}_{\text{mirror}}^{-1}\tilde{H}^T\tilde{T}_t^{-1}\tilde{\mathbf{s}} = \begin{pmatrix} -x(\sin(\alpha_u)\sin(\alpha_t)+\cos(\alpha_u)\cos(\alpha_t)\cos(\theta)) - y(\sin(\alpha_u)\cos(\alpha_t)-\cos(\alpha_u)\sin(\alpha_t)\cos(\theta)) - z\cos(\alpha_u)\sin(\theta) \\ x(\cos(\alpha_u)\sin(\alpha_t)-\sin(\alpha_u)\cos(\alpha_t)\cos(\theta)) + y(\cos(\alpha_t)\cos(\alpha_u)+\sin(\alpha_u)\sin(\alpha_t)\cos(\theta)) - z\sin(\alpha_u)\sin(\theta) \\ x\cos(\alpha_t)\sin(\theta) - z\cos(\theta) - y\sin(\alpha_t)\sin(\theta) \\ 1 \end{pmatrix}. \tag{50}$$

Since V' is a delta distribution concentrated in the XY-plane, its integral along Z coincides with the value of V' when the third coordinate is 0, that is

$$x\cos(\alpha_t)\sin(\theta) - z\cos(\theta) - y\sin(\alpha_t)\sin(\theta) = 0,$$

or, what is the same, when

$$z = x\cos(\alpha_t)\tan(\theta) - y\sin(\alpha_t)\tan(\theta),$$

which is exactly the same condition as in the previous proof. Substituting this value into Eqs. (50) and (48), it can be checked that

$$I_t(\tilde{\mathbf{s}}) = \int_{-\infty}^{\infty} V'(\tilde{E}_{\text{mirror}}^{-1}\tilde{H}^T\tilde{T}_t^{-1}\tilde{\mathbf{s}})dz = V'(\tilde{H}_0^T(\tilde{H}_0\tilde{E}_{\text{mirror}}\tilde{H}_0^T)^{-1}\tilde{T}_t^{-1}\tilde{\mathbf{s}}). \tag{51}$$

According to Eq. (49) we have

$$\begin{aligned} I_u(\tilde{M}(\tilde{H}_0\tilde{E}_{\text{mirror}}\tilde{H}_0^T)^{-1}\tilde{T}_t^{-1}\tilde{\mathbf{s}}) &= V'(\tilde{H}_0^T\tilde{M}\tilde{M}(\tilde{H}_0\tilde{E}_{\text{mirror}}\tilde{H}_0^T)^{-1}\tilde{T}_t^{-1}\tilde{\mathbf{s}}) \\ &= V'(\tilde{H}_0^T(\tilde{H}_0\tilde{E}_{\text{mirror}}\tilde{H}_0^T)^{-1}\tilde{T}_t^{-1}\tilde{\mathbf{s}}) \\ &= I_t(\tilde{\mathbf{s}}), \end{aligned} \tag{52}$$

which concludes the proof.

6 Supplementary figures

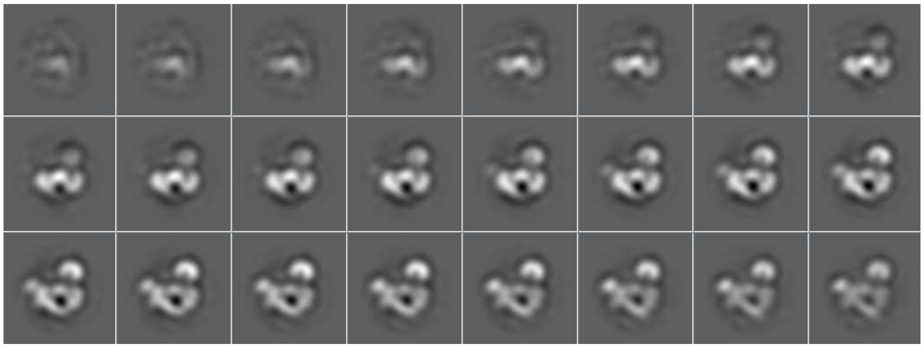

Figure 1: Control map: Central slices of the control map of C3b computed from 32,595 images and a 3D classification procedure (see main text for details).

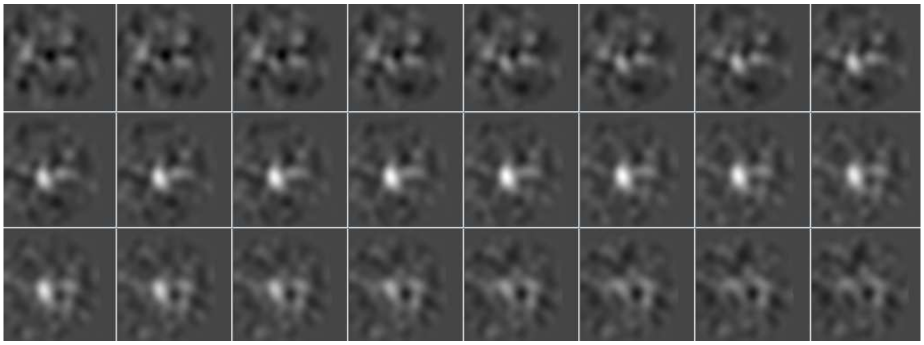

Figure 2: Standard RCT workflow for small-sized 2D classes (20 image pairs): Central slices of the structure of C3b using standard RCT, as implemented in Spider. Compare them to Suppl. Fig. 1.

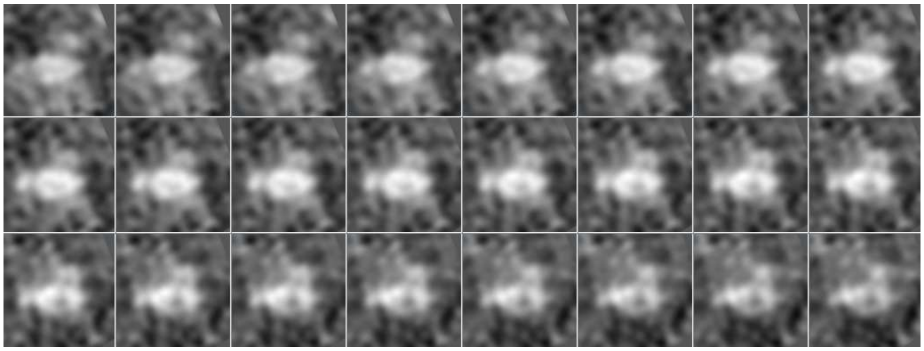

Figure 3: Modified RCT workflow for small-sized 2D classes (13 image pairs): Central slices of the structure of C3b using modified RCT, as implemented in Xmipp. Compare them to Suppl. Fig. 1.

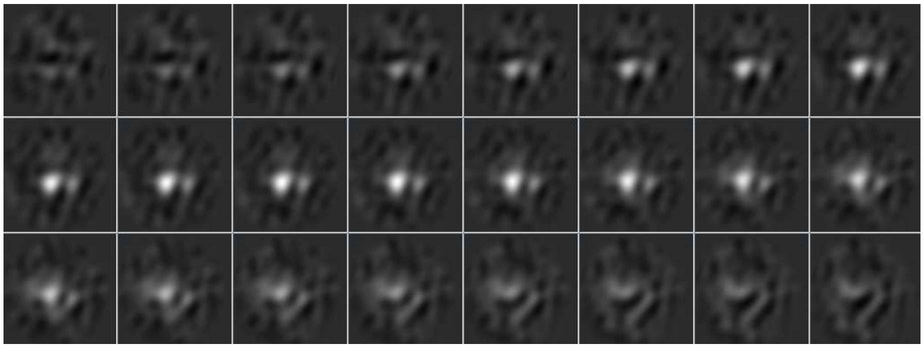

Figure 4: Standard RCT workflow for mid-sized 2D classes (79 image pairs): Central slices of the structure of C3b using standard RCT, as implemented in Spider. Compare them to Suppl. Fig. 1.

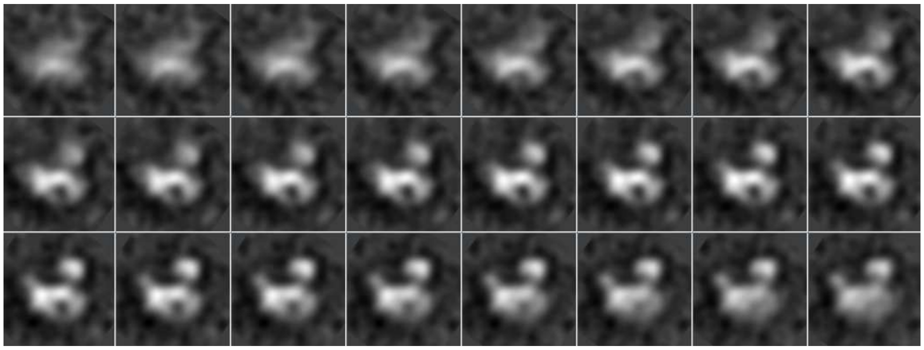

Figure 5: Modified RCT workflow for mid-sized 2D classes (61 image pairs): Central slices of the structure of C3b using modified RCT, as implemented in Xmipp. Compare them to Suppl. Fig. 1.

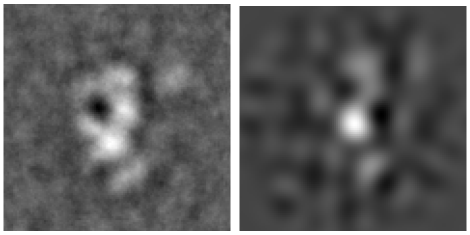

Figure 6: Left: Class average of 20 untilted projections calculated with Spider. Right: Central slice of the corresponding RCT reconstruction calculated with standard RCT, as implemented in Spider.

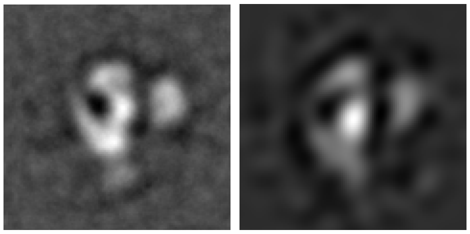

Figure 7: Left: Class average of 79 untilted projections calculated with Spider. Right: Central slice of the corresponding RCT reconstruction calculated with standard RCT, as implemented in Spider.

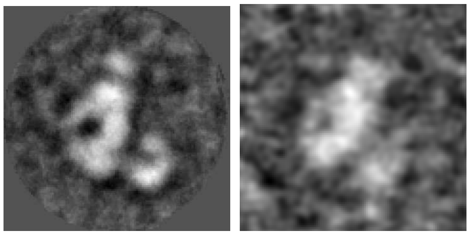

Figure 8: Left: Class average of 13 untilted projections calculated with Xmipp. Right: Central slice of the corresponding RCT reconstruction calculated with modified RCT, as implemented in Xmipp.

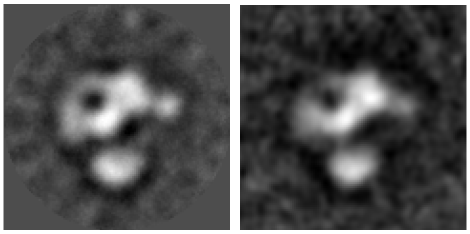

Figure 9: Left: Class average of 61 untilted projections calculated with Xmipp. Right: Central slice of the corresponding RCT reconstruction calculated with modified RCT, as implemented in Xmipp.

Bibliography

References

[Guckenberger(1982)] Guckenberger, R., 1982. Determination of a common origin in the micrographs of titl series in three-dimensional electron microscopy. Ultramicroscopy 9, 167–174.

[Hauer et al.(2013)] Hauer, F., Gerle, C., Kirves, J.-M., Stark, H., Feb 2013. Automated correlation of single particle tilt pairs for random conical tilt and orthogonal tilt reconstructions. J Struct Biol 181 (2), 149–154.
URL `http://dx.doi.org/10.1016/j.jsb.2012.10.014`

[Hegerl et al.(1991)] Hegerl, R., Pfeifer, G., Pühler, G., Dahlmann, B., Baumeister, W., May 1991. The three-dimensional structure of proteasomes from thermoplasma acidophilum as determined by electron microscopy using random conical tilting. FEBS Lett 283 (1), 117–121.

[Radermacher(1988)] Radermacher, M., 1988. Three-Dimensional reconstruction of single particles from random and nonrandom tilt series. J. Electron Microscopy Technique 9, 359–394.

[Scheres et al.(2005)] Scheres, S. H. W., Valle, M., Núñez, R., Sorzano, C. O. S., Marabini, R., Herman, G. T., Carazo, J. M., 2005. Maximum-likelihood multi-reference refinement for electron microscopy images. J. Molecular Biology 348, 139–149.

[Shatsky et al.(2014)] Shatsky, M., Arbelaez, P., Han, B. G., Typke, D., Brenner, S. E., Malik, J., Glaeser, R. M., Jul 2014. Automated particle correspondence and accurate tilt-axis detection in tilted-image pairs. J. Structural Biology 187 (1), 66–75.
URL `http://dx.doi.org/10.1016/j.jsb.2014.03.017`

[Sorzano et al.(2010)] Sorzano, C. O. S., Bilbao-Castro, J. R., Shkolnisky, Y., Alcorlo, M., Melero, R., Caffarena-Fernández, G., Li, M., Xu, G., Marabini,

R., Carazo, J. M., 2010. A clustering approach to multireference alignment of single-particle projections in electron microscopy. J. Structural Biology 171, 197–206.

[Sorzano et al.(2004)] Sorzano, C. O. S., Marabini, R., , Velázquez-Muriel, J., Bilbao-Castro, J. R., Scheres, S. H. W., Carazo, J. M., Pascual-Montano, A., 2004. XMIPP: A new generation of an open-source image processing package for electron microscopy. J. Structural Biology 148, 194–204.

[Voss et al.(2009)] Voss, N. R., Yoshioka, C. K., Radermacher, M., Potter, C. S., Carragher, B., May 2009. Dog picker and tiltpicker: software tools to facilitate particle selection in single particle electron microscopy. J Struct Biol 166 (2), 205–213.

[Zampighi et al.(2004)] Zampighi, L. M., Kavanau, C. L., Zampighi, G. A., 2004. The kohonen self-organizing map: a tool for the clustering and alignment of single particles imaged using random conical tilt. J. Structural Biology 146, 368–380.

[Zheng et al.(2007)] Zheng, S. Q., Kollman, J. M., Braunfeld, M. B., Sedat, J. W., Agard, D. A., Jan 2007. Automated acquisition of electron microscopic random conical tilt sets. J. Structural Biology 157 (1), 148–155.
